# Application of decellularized tissue for soft-hard interregional regeneration

**DOI:** 10.3389/fbioe.2025.1394714

**Published:** 2025-04-16

**Authors:** Mika Suzuki, Tsuyoshi Kimura, Yoshihide Hashimoto, Akio Kishida

**Affiliations:** ^1^ Laboratory for Biomaterials and Bioengineering, Institute of Science Tokyo, Tokyo, Japan; ^2^ Department of Biomedical Engineering, Toyo University, Saitama, Japan; ^3^ Joining and Welding Research Institute, Osaka University, Osaka, Japan

**Keywords:** decellularized tissue, soft-hard interregional tissue, tissue regeneration, tendon, ligament, periodontal ligament (PDL), extracellular matrix (ECM)

## Abstract

Decellularized tissue refers to extracellular matrix (ECM) derived from living tissue by removing the cellular components and is used for tissue regeneration. Various decellularized tissue sheets and powders, such as the dermis, urinary bladder matrix, and small intestinal submucosa, have been clinically used as covering and prosthetic materials. Moreover, there is growing interest in the use of decellularized tissue for soft-hard interregional tissue regeneration, including in the ligament-bone, tendon-bone, and periodontal ligament-bone interfaces. The focus in these applications lies in the mechanical properties of the decellularized tissue. Decellularized ligaments and tendons have been developed using various decellularization methods, with a focus on maintaining their shape and mechanical properties, and have been applied orthotopically or ectopically to ligaments and tendons. In the ligament-bone interface, it is suggested that decellularized ligament and tendon are regenerated through the migration and rearrangement of host cells, which is referred to as “*in situ* tissue regeneration.” It is also proposed that decellularized tissue can be used to prepare the complex structure of soft-hard interregional tissue, which consists of an ECM and cell populations with gradual change. In this case, the decellularized soft tissues of ligaments, tendons, pericardium, and others are fabricated and modified with hard tissue components to mimic the gradual structure of soft-hard interregional tissue. In this review, we present a detailed discussion of the regeneration of soft-hard interregional tissue using decellularized tissue.

## 1 Introduction

Decellularized tissue is obtained by removing the cellular components in living tissues. Decellularized tissues are composed of extracellular matrices (ECMs), such as collagen and glycosaminoglycans (GAGs), which maintain the complex three-dimensional structure of living tissues. They are also used as alternative materials and scaffolds for tissue regeneration. At present, many decellularized tissue products are available in the United States and European markets (Parmaksiz et al., 2016; Capella-Monsonis et al., 2021). A wide variety of these products are derived from human, porcine, bovine, and other allogeneic and xenogeneic animal sources (Table 1). Many of these products are fabricated into sheets and powders and are used as alternative materials in a variety of applications, such as tissue coverings and fillings. It is believed that various bioactive substances contained in decellularized tissues act on them to induce tissue regeneration. However, the types of bioactive substances that are present in decellularized tissue remains unclear. Many studies have been conducted to take advantage of this feature to regenerate tissues by implanting ECMs as scaffolds in various fields, including orthopedics and dentistry, which are the main fields associated with soft-hard interregional tissue regeneration. In the field of orthopedics, the regeneration of tendons and ligaments has been researched using decellularized tendons and ligaments, including ligament–bone and tendon-bone regeneration. Several protocols that implement chemical and physical decellularization methods have been proposed, and their biocompatibility and functional regeneration have been investigated. Recently, it has been proposed that decellularized tissue can be used for soft-hard interregional tissue regeneration. In the ligament-bone interface, it is suggested that decellularized tendon and ligament tissues can be inserted into bone and regenerated through the migration and rearrangement of host cells, which is referred to as “*in situ* tissue regeneration”. In addition, to enhance the recruitment of host cells to decellularized tissue, various attempts, such as fabrication, remodeling, and modification of decellularized tissue, are being made. In the dental field, regeneration of the periodontal ligament (PDL) is focused on soft-hard interregional tissue. In addition to the concept of *in situ* tissue regeneration, in which a decellularized mandibular bone with a PDL matrix recruits host cells and is regenerated, the use of a decellularized cell sheet and a decellularized membrane tissue as the periodontal ligament has been proposed. Herein, we discuss the various methods used to obtain decellularized tissue, the properties of decellularized tissue, fabrication of decellularized tissue and potential applications in orthopedics and dentistry.

**TABLE 1:** Examples of commercially available decellularized tissue products.

Source	Application	Products (manufacture)
Human dermis	Soft tissue, Tendon, Uterus, Breast, etc.	AlloDerm (LifeCell), Allo Max(Becton, Dickinson and Company), Allo Patch (MTF, Edison), Arthro Flex (Arthrex, LifenetHealth), Axis (Coloplast), Cortiva, Matrix HD (RTI Surgical), Flex HD (MTF, Edison), GRAFTJACKET (LifeCell), OrACELL (Life net Health), SureDerm (Hans Biomed Corp)
Human fascia lata	Urethra	Suspend (Coloplast)
Human pericardium	Ophthalmology	IOP Patch (IOP)
Human aorta	Heart	Cryo Patch (CryoLife)
Human heart valve	Heart valve	Cryo Valve (CryoLife)
Human bone, cartilage	knee joint	AlloWedge, Elemax, Bioadapt, Map3 (RTI Surgical)
Porcine dermis	Soft tissue	Fortiva(RTI Surgical), Permacol Surgical Implant (Medtronic), Strattice (Allergan), XenMatrix (Becton, Dickinson and Company), CollagenRepairPatch (Zimmer)
Porcine heart valve	Heart valve	Epic, Trifecta (Abbott), Freestyle, HancockⅡ, Mosaic (Medtronic)
Porcine SIS	Soft tissue, Pericardium	Oasis, Biodesign (Cook Medical), CorMatrix ECM (Aziyo)
Porcine UBM	Soft tissue	MatriStem, Acell Vet (Acell)
Porcine cornea	Cornea	Acornea
Bovine dermis	Soft tissue	PriMatrix, SurgiMend, TissueMend (Integra Life science)
Bovine pericardium	Dental, cornea, Heart valve, Soft tissue	CopiOs (ZimVie), Lyoplant(B.Braun Melsungen), Perimount(Edwards Lifesciences), TutoPatch(RTI Surgical)

## 2 Decellularization methods

**FIGURE 1:**
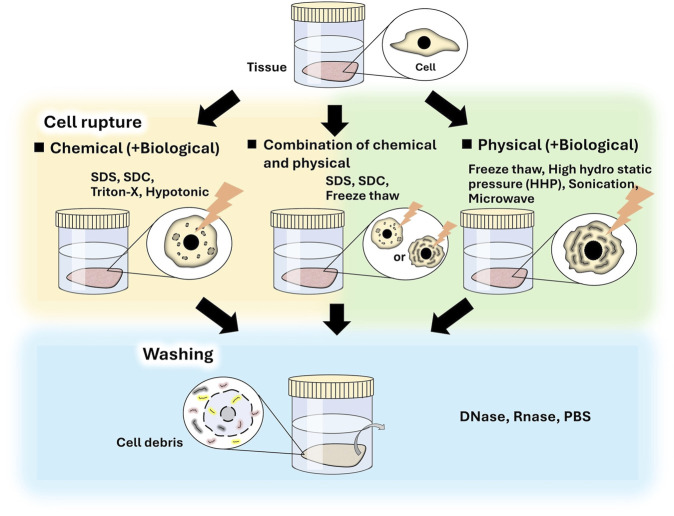
Different decellularization methods.

Decellularization methods usually include two main processes: cell destruction and washing of cell debris. Many decellularization methods have been proposed and are either classified as chemical or physical in the viewpoint of cell destruction process (Figure 1). Biological agents such as trypsin, dispase, and nuclease, are also used to enhance decellularization and to remove cell residues. Both methods include biological processes using nuclease to remove cell residues.

Chemical decellularization is a major method. In these methods, tissue is immersed in solutions, such as surfactant, or hypotonic and hypertonic solutions, and then washed to remove cell debris and surfactant. Various surfactants, such as sodium dodecyl sulfate (SDS) (Mirsadraee, et al., 2007; Willemse et al., 2020; Reing et al., 2010), Triton X-100^®^ (Jeong et al., 2021; Reing et al., 2010; Tiemann et al., 2020; Sulivan et al., 2012), and sodium deoxy cholate (SDC) (Lichtenberg et al., 2006; Cabotari et al., 2010; McCrary et al., 2020), have been used for tissue decellularization (Gilbert et al., 2006; Zhang et al., 2021; Nakamura et al., 2017). In these methods, cells are effectively removed; however, in many cases, the tissue is damaged because the surfactant dissolves cellular membranes and ECM components, including collagen and glycosaminoglycan, in the tissue (Wu et al., 2015; Kobayashi et al., 2020; Crapo et al., 2011). Therefore, using an appropriate type and concentration of surfactant and controlling the treatment time is important to obtain decellularized tissue for specific purposes. SDS is the most commonly used surfactant for producing decellularized tissue. Although efficient decellularization is achieved by SDS treatment, the ECMs are often severely damaged (Faulk et al., 2014). The structure of decellularized tissue influences cellular functions, such as adhesion, morphology, growth, and differentiation, during tissue regeneration (Wei et al., 2020; Agmon et al., 2016; Negishi et al., 2017a); therefore, the decellularization protocol should be carefully considered. In addition, it is necessary to thoroughly wash SDS-treated tissue due to the cytotoxicity of SDS. SDC and Triton X-100 are often used for decellularization and facilitate relatively mild decellularization owing to their low surfactant activities. In addition, their cytotoxicity is lower than that of SDS. A combination of detergents can effectively decellularize tissues. For example, tissues treated with SDS can be treated with Triton X-100 to remove residual SDS (Willemse et al., 2020, Sulivan et al., 2012).

Physical decellularization methods, such as freeze-thawing (Watanabe et al., 2019; Burk et al., 2014), supercritical carbon dioxide (CO_2_) (Sawada et al., 2008; Casali et al., 2018; Kim et al., 2021), and high hydrostatic pressure (HHP) (Funamoto et al., 2010; Mahara et al., 2015; Nakamura et al., 2017), are used to destroy cells and cell debris are subsequently washed away. Special equipment is often required to destroy cells. During the washing process, nucleases are used to enhance the removal of DNA and RNA from cell debris because the diffusion of digested DNA is increased. The conditions of the freeze–thaw method, such as time, temperature, and cycle, are specifically adjusted for each tissue sample. The effect of decellularization generally increases with an increase in the number of freeze–thaw cycles. Freezing and freeze-thawing of tissues have been reported to affect biological responses (Kobayashi et al., 2020), and the freeze-thaw decellularization method should be carefully considered. In the supercritical CO_2_ method, cellular components are extracted using supercritical fluid. In supercritical CO_2_ fluid, which is generally applied under mild conditions (37°C, low pressure), low reactivity to polar components, such as proteins and polysaccharide chains, hinders biomolecules. In addition, supercritical CO_2_ molecules exhibit properties such as liquid-like solute solubility and gas-like diffusion, which increases the tissue penetration ability of CO_2_ and the solubility of nonpolar molecules in tissues (Sawada et al., 2008, Seo et al., 2018). In HHP decellularization methods, cells are destroyed using high hydrostatic pressurization (200 MPa); the denaturation of proteins is induced by hydrostatic pressures of more than 300 MPa. The effect of decellularization differs depending on the tissue and the conditions, such as pressure, time, and temperature, which are adjusted for each tissue type (Sasaki et al., 2009; Funamoto et al., 2010; Hashimoto et al., 2010). After cell destruction, the cell debris are removed by washing. The washing time is generally related to the size, thickness, and volume of the tissue, and long-term washing is required to remove cell debris from deep tissue sites. In this method, the structure of the decellularized tissue generally remains unchanged compared to that of chemically-decellularized tissue, in which the ECM is dissolved and washed away (Kobayashi et al., 2020; Negishi et al., 2017a).

A combination of chemical and physical methods can effectively decellularize tissues. After cell rupture using freeze-thawing, cellular debris can be removed using a surfactant, which is most effective when used at a low concentration for a short period (Kheir et al., 2011; Herbert et al., 2015; Jones et al., 2017; Whitaker et al., 2019; Edwards et al., 2019). Cells in the tissue can also be destroyed using a hypotonic solution or surfactant solution and then effectively removed using ultrasonic treatment or supercritical CO_2_ treatment (Casali et al., 2018). In view of the above, it is important to consider the purpose of the research when selecting an appropriate decellularization method; tissues vary from cell-based tissues with a high cell density to ECM-based tissues with high ECM density, which are of various sizes, thicknesses, and volumes.

Table 2 presents the different methods of decellularization used for different tissue types, such as ligaments, tendons, cartilage, and joints. Decellularization of tendons, ligaments, cartilage, and joints at various sites, such as the shoulder, knee, and ankle, has been attempted in a variety of species, including humans, pigs, rabbits, dogs, and rats. Decellularization has been performed using chemical and physical methods, and combinations thereof. Chemical methods are often used for the preparation of decellularized tendons and ligaments, and surfactants are often the solution of choice. SDS, Triton X-100, and SDC are generally used as surfactants, and the type, combination, and concentration of surfactants vary and are adjusted for each tissue type. Physical methods, such as freeze-thaw and microwave methods, have also been used to decellularize tendons and ligaments. When a combination of chemical and physical methods is used, cells are destroyed by freezing, thawing, or ultrasound, followed by surfactant or enzyme treatment. There have been many reports of decellularization using surfactants; however, recently, there has been a trend toward decellularization using a combination of freeze-thawing and low concentrations of surfactants.

**TABLE 2:** Decellularization methods for tendon, ligament, cartilage, and joint.

Method	Tissue	Tissue source	Note	References
Chemical	Bone-anterior cruciate ligament-bone	Porcine	SDS, Triton X-100	Harrison and Gratzer (2005); Woods and Gratzer (2005)
Chemical	Anterior cruciate ligament	Porcine	SDS, Triton X-100	Gratzer et al. (2006); MacLean and Gratzer (2011)
Chemical	Anterior cruciate ligament	Porcine	Triton X-100, SDS, SDC	Vavken et al. (2009); Yoshida et al. (2012)
Chemical	Flexor tendon	Human	SDS	Raghavan et al. (2012)
Chemical	Finger proximal interphalangeal joint	Human	SDS	Endress et al. (2012)
Chemical	Dorsal scapholunate ligament	Human	SDS	Endress et al. (2012)
Chemical	Bone-tendon	Human	SDS	Farnebo et al. (2014)
Chemical	Semitendinous tendon	Rabbit	Triton X-100, Trypsin	Lu et al. (2019)
Chemical	Hamstrings tendon	Rabbit	Triton X-100, Trypsin	Li et al. (2017)
Combination	Superflexor tendon	Porcine	Freeze- thaw/SDS	Hexter et al. (2020); Whitaker et al. (2019); Jones et al. (2017); Herbert et al. (2015); Edwards et al. (2019)
Combination	Bonecartilage	Porcine	Freeze- thaw/SDS	Kheir et al. (2011)
Combination	Knee joint	Rabbit	Freeze- thaw/SDS	Zhang et al. (2021)
Combination	Patella tendon	Porcine	SDS/Ultrasonication	Ingham et al. (2007)
Combination	Tibialis tendon	Porcine	Ultrasonication/Trypsin	Lee et al. (2018)
Physical	Tendon	Bovine	Microwave	Itoh et al. (2022)
Physical	Flexor digitorum superficialis tendon	Rabbit	Freeze-thaw	Liu et al. (2017)
Physical	Flexor digitorum superficialis tendon	Dog	Freeze-thaw	Lu et al. (2019)

## 3 Characterization of decellularized tissue

Decellularized tissue is often evaluated using residual DNA quantification, residual ECM quantification, and histological observation. The amount of residual DNA in the decellularized tissue is measured, and 50 ng/mg tissue can be used as a standard for decellularization, as proposed by Crapo et al. (Crapo et al., 2011). The length of the residual DNA is also investigated, and a DNA length of less than 200 bp is required as the standard for decellularization (Crapo et al., 2011). Methods for quantifying residual DNA include the measurement of absorbance, which quantifies the total amount of DNA extracted from the decellularized tissue, and double-stranded DNA quantification using a fluorescent intercalator (e.g., PicoGreen). Residual DNA can be evaluated using a tissue fragment image stained with a fluorescent intercalator, such as Hoechst. The amount of residual ECM, such as collagen and sulfated GAGs, is also quantified. Methods for the determination of residual collagen include hydroxyproline and triple amino acid (glycine-x-y: GXY) determination, which are typical amino acid residues and sequences of collagen. The type of collagen, such as types I, II, II, and VI, is also evaluated because the ratio of collagen types differs among tissue types. Sulfated GAG can also be used to quantify the residual GAGs. The structure of the decellularized tissue is evaluated by hematoxylin and eosin (H&E) staining and immunostaining. Also, the remained growth factors and ECM contents in decellularized tissues are investigated. Several growth factors, such as basic fibroblast growth factor, vascular endothelial growth factor and transforming growth factor beta, are remained in decellularized. The amount of growth factors was depended on used tissues and decellularization methods (Badylak, 2007; Hanai et al., 2020; Mineta et al., 2023). Recently, the decellularized tissue is analyzed by proteomics (Bonvillain et al., 2012; Hill et al., 2015; Calle et al., 2016; Thomas-Potch et al., 2018; Diedrich et al., 2024; Biehl et al., 2023). The removal of intercellular proteins and the remaining of proteoglycans and glycoproteins could be evaluated by proteomic analysis (Hill et al., 2015). Also, tissue-specific proteins were detected in detail (Diedrich et al., 2024).

For decellularized tendons and ligaments, it is important that the amount of DNA remaining meets the criteria for decellularization in any chemical, physical, or combined method. Decellularization is performed to meet these criteria. In rare cases, the criteria are not met, but a significant reduction in the amount of residual DNA in decellularized tissue compared to that in untreated tissue has been demonstrated (Balogh et al., 2016). The amount of ECM remaining after decellularization is quantified, and the amount of collagen and GAG varies depending on the tissue type and the decellularization method implemented. It was previously reported that SDS decellularization of the patellar tendon resulted in no difference in collagen or sGAG levels before and after decellularization (Ingram et al., 2007). In addition, freeze-thawing/SDS decellularization of superflexor tendon (SFT) resulted in no difference in the collagen residues before and after decellularization; however, the level of sGAG residues decreased due to decellularization (Jones et al., 2017). After decellularization of dog superficial digital flexor tendons (SDFTs) and deep digital flexor tendons (DDFTs) in a hypertonic solution with nonionic detergent, the amount of residual DNA was reduced compared to native tissues, whereas the sGAG, collagen, and protein levels were maintained (Balogh et al., 2016). As described above, the composition of the remaining ECM varies depending on the type of tissue and decellularization method used; therefore, method selection is highly dependent on the purpose of the research. Proteomics of decellularized tendon and ligament.

## 4 Mechanical properties of decellularized tissue

The mechanical properties of decellularized tissues are among the most important parameters that must be considered before their implantation as a replacement graft material. The mechanical properties of decellularized tissue vary depending on the tissue used, the decellularization method, and the chemical properties of the decellularized tissue. The mechanical properties of the tissue, such as elastic modulus, tensile strength, and failure strain, are generally reduced by decellularization (Wu et al., 2015; Xu et al., 2014; EL-Husseiny et al., 2023). The degree of the reduction in mechanical properties depends on the type of tissue and the decellularization method used. Tissues with a low cell density and high ECM density, such as ligament, tendon, and pericardium, generally retain their mechanical properties (Suzuki et al., 2022; de Lima Santos et al., 2020; Ning et al., 2012). In contrast, tissues with an intermediate cell density and ECM density, such as the aorta and dermis, experience a mild reduction in their mechanical properties (Wu et al., 2015). Tissues with a high cell density and low ECM density, such as lung and liver tissue, exhibit the most significant reduction in mechanical properties (Petersen et al., 2012). The mechanical properties of decellularized tissue are significantly affected by the decellularization method in relation to its chemical properties, such as the amount of ECM and the degree of denaturation. The mechanical properties of surfactant-decellularized tissue exhibit more significant reductions than that of HHP-decellularized tissue because structural ECMs, such as collagen and elastin, are removed. We previously compared the mechanical properties of a HHP-decellularized aorta and SDS-decellularized aorta. The HHP-decellularized aorta was mechanically and structurally similar to the native aorta; however, the structure of the SDS-decellularized aorta was disordered, resulting in poor mechanical properties (Wu et al., 2015).

As described in Section 3, decellularization methods are known to affect the composition of the tissue, which in turn, affects the mechanical properties of the decellularized tissue. Compared with physical decellularization methods, chemical decellularization methods exhibit a higher ECM removal capacity, which may result in reduced mechanical properties. With regards to tendons and ligaments that are decellularized by various methods, differences in mechanical properties, such as ultimate tensile strength (UTS), failure strain (FS), and elastic modulus (E), have been investigated before and after decellularization. The mechanical properties of decellularized tendons were summarized in Table 3. Because the shapes of tendons and ligaments vary according to the species and site, measurements are made using custom-made clamps according to the shape of the tissues used. It has been reported that there is no difference in the mechanical properties (UTS, FS, and E) of the rat bone-Achilles tendon, rabbit semitendinosus tendon, and canine tendon (Farnebo et al., 2014; Dong et al., 2015; Balogh et al., 2016). In addition, although no difference in the mechanical strength of the porcine superflexor tendon was observed before and after surfactant decellularization (Jones et al., 2017), a significant decrease in breaking stress was observed after a combination of freeze-thawing and SDS treatment in a porcine superflexor tendon; however, no other significant differences were observed (Edwards et al., 2019). This suggests that it is necessary to precisely measure various mechanical parameters because the effects of the decellularization method vary depending on the tissue and species. The mechanical properties under dynamic physiological loading conditions were measured using special equipment. Significant reductions in the dynamic modulus, storage modulus, and loss modulus were observed at all measured frequencies. However, there was no significant difference in damping ability (tan δ), indicating no change in force transfer efficiency (Edwards et al., 2019). Dynamic stiffness was found to increase significantly with the number of cycles; after 1,000 cycles of loading, there was no significant interaction between graft size and cycles elapsed (Whitaker et al., 2019). By measuring mechanical properties under conditions similar to actual ligament movement, it is possible to understand the characteristics of decellularized tendon and ligament in detail. Also, the dynamic physiological measurement of decellularized tendon and ligament is important to use them as medical device in the view of the regulatory science.

**TABLE 3:** Mechanical property of decellularized tendons described in Section 4.

Tissues			Load at Ultimate Failure	Ultimate tensile strain (UTS)	Failure strain (FS)	Stiffness	Elastic modulus (E)	Ref.
Canine tendon		Native DDFT (n=5)	2014.3 ± 229.5 N			473.7± 146.3 (N/m)	136.4±52.9 (N/mm^2^)	Balogh et al. (2016)
		Decellularized DDFT (n=5)	1954.5 ± 620.1 N			445.5± 124.3 (N/m)	114.8±37.2 (N/mm^2^)	
		Native SDFT (n=6)	1721.3 ± 729.9 N			420.9± 110.4 (N/m)	101.3±24.0 (N/mm^2^)	
		Decellularized SDFT (n=6)	1594.0 ± 368.7 N			413.2± 154.7 (N/m)	129.7±49.3 (N/mm^2^)	
Rabbit Semitendinosus tendon (ST)		Double-strand fresh-frozen STs	185.95 ±7.91 N			45.99± 5.49 (N/m)		Dong et al. (2015)
		Double-strand decellularized STs	200.39 ± 22.11 N			44.26± 2.96 (N/m)		
Porcine super flexor tendon (pSFT)		Native		52.5± 5.9 MPa	0.33± 0.05 (mm/mm)		234.2± 51.3 (MPa)	Jones et al. (2017)
		Acellular		61.8± 10.3 MPa	0.29± 0.04 (mm/mm)		294.1 ±61.9 (MPa)	
Rat (SD) bone-Achilles tendon (AT)	Before implantation	Untreated control	75.7 ± 18.5 N	10.2± 4.9 MPa				Farnebo et al. (2014)
		Decellularized bone-AT	69.5 ± 6.0 (NS) N	10.7 ±3.0 (NS) MPa				
	After implantation	Untreated grafts (2wk)	23.3± 9.1 N	1.2± 0.6 MPa				
		Decellularized grafts (2wk)	31.7± 7.4 N	1.9± 0.6 MPa				
		Untreated grafts (4wk)	22.7± 9.6 N	1.1± 0.5 MPa				
		Decellularized grafts (4wk)	46.9± 12.7 N	2.3± 0.7 MPa				

As described above, the tendons and ligaments of various species have been decellularized using chemical methods (mainly surfactants), and their mechanical properties have been evaluated, often exhibiting no reductions in mechanical strength due to decellularization. In contrast, the use of surfactants for the decellularization of soft tissues, such as blood vessels and skin, has been reported to reduce mechanical strength (Wu et al., 2015; Zhang et al., 2018). This difference may be because the main components of tendons and ligaments are composed of collagen fibers. Interestingly, rat tendons with and without surfactant decellularization were implanted allogenetically, and the mechanical properties, such as ultimate failure load, ultimate tensile stress, and stiffness of the decellularized tendons, were more favorable than those of untreated tendons, although their mechanical properties were reduced by transplantation (Farnebo et al., 2014) (Bottom of Table 3). The mechanical reduce may be caused by the degradation of decellularized tissue *in vivo*. So, to hinder the *in vivo* degradation of decellularized tissue and maintain mechanical strength, the use of cross-linking agents, such as naringin, has been also proposed (Cheng et al., 2023). For the transplantation of decellularized tissue, the tissue reconstruction including degradation and recellularization occur and the further investigation for biological reconstruction processes is needed using various decellularized tissues having different components, histological structures which prepared by various decellularization methods.

## 5 Biocompatibility of decellularized ligaments and tendons

Decellularized tissue is used as a scaffold material, either orthotopically or ectopically. Orthotopic application is a method in which the same tissue as the implant site is decellularized and implanted orthotopically, and the tissue is reconstructed (orthotopic tissue regeneration). Ectopic application occurs when decellularized tissue, which is different from the implant site, is used and reconstructed into a tissue that is appropriate for the implant site (ectopic tissue regeneration). Decellularized tissue acts as a scaffold and cells around the implant site regulate tissue reconstruction, which is suitable for the implant site. Many decellularized tissue products are applied to both orthotopic and ectopic sites to promote tissue reconstruction at the implant site, regardless of the origin of the decellularized tissue. Although the reason for this is still not clear, it is suggested that the bioactive substances in decellularized tissue, such as growth factors, small vesicles, as well as its histological structure and mechanical properties, affect the reconstruction of tissue, including immunological reactions and cellular behaviors.

To use decellularized ligaments and tendons orthotopically and ectopically, their biocompatibility must be evaluated *in vitro* and *in vivo*. Biocompatibility is examined in terms of cell affinity (cell adhesion and cytotoxicity) in in vitro cell cultures and viability and cell invasion in in vivo transplantation models using small to large animals. The adhesive and proliferative properties of fibroblasts are also investigated to determine their *in vitro* cell affinity. Decellularized tendons have been prepared using different surfactants that exhibit different cell-adhesion properties (Harrison and Gratzer, 2005). The seeding of cells on a decellularized porcine patella tendon also revealed cell adhesion and proliferation on the surface, but no cell infiltration into the interior (center) was observed, even after 6 weeks of culture (Ingram et al., 2007). Cell seeding on a decellularized porcine anterior cruciate ligament also exhibited some internal infiltration of fibroblasts, but only in 11%–19% of fresh ligaments (MacLean and Gratzer, 2011). Internal infiltration of fibroblasts was only partially clustered when cell seeding on a TritonX-100-decellularized anterior cruciate ligament (Vavken et al., 2009). During *in vitro* cell seeding, decellularized tissue did not exhibit cytotoxicity. Furthermore, fibroblast adhesion and proliferation, but not internal infiltration, were observed. In contrast, the application of fibroblast chemotactic factors, such as basic fibroblast growth factor (bFGF), to decellularized tendons did not result in sufficient cell infiltration (Harrison and Gratzer, 2005), suggesting that the high fiber density of tendons and ligaments inhibits cell infiltration into the interior *in vitro*.

Ligaments and surrounding tissues reconstructed using decellularized tendons do not exhibit necrosis or inflammation (Lee et al., 2018). A comparison of a rabbit anterior cruciate ligament (ACL) with and without decellularized allografts revealed better fibroblast infiltration, vascularization, connective tissue formation, and neoplastic bone formation in the decellularized tissue (Dong et al., 2015). In contrast, in a report of decellularized Achilles tendons subcutaneously transplanted into rats, angiogenesis was observed around the sample, but the sample tended to degrade, suggesting that uncross-linked Achilles tendons are not suitable for ACL reconstruction (Cheng et al., 2023). Although there is some concern regarding the tendency of decellularized tissue to degrade before tissue reconstruction, many *in vivo* transplant studies have reported that decellularized tissue infiltrates cells and exhibits better cell affinity than the target group. In rat tendon grafts, increased B cell and macrophage infiltration was observed in both the capsule surrounding the tendon-bone interface and in the tendon parenchyma of untreated controls at 2 and 4 weeks after implantation (Farnebo et al., 2014). In bovine decellularized tendons transplanted into rat ACLs through xenografting, M1 macrophage accumulation was observed around the autologous tendon, indicating inflammation, whereas M2 macrophages accumulated around the decellularized tendon, indicating tissue regeneration. Decellularized tendons are thought to induce an M2-dominant host response and induce cellular infiltration into xenografts compared to autografts. Cells in the grafts of the intra-articular and intratibial regions were comparable to those in the native ACL in both groups after 4 and 8 weeks, respectively. The number of M2 macrophages in the intra-articular and intra-tibial tunnel grafts was highest at week 4 in both groups (Itoh et al., 2022). These reports suggest that xenografts and allografts of decellularized tissues are acceptable both *in vitro* and *in vivo*. Research on the biological response to decellularized tendons and ligaments has just begun, and is important to determine which tissue should be used at the target site.

## 6 Fabrication and modification of decellularized tissues

**FIGURE 2:**
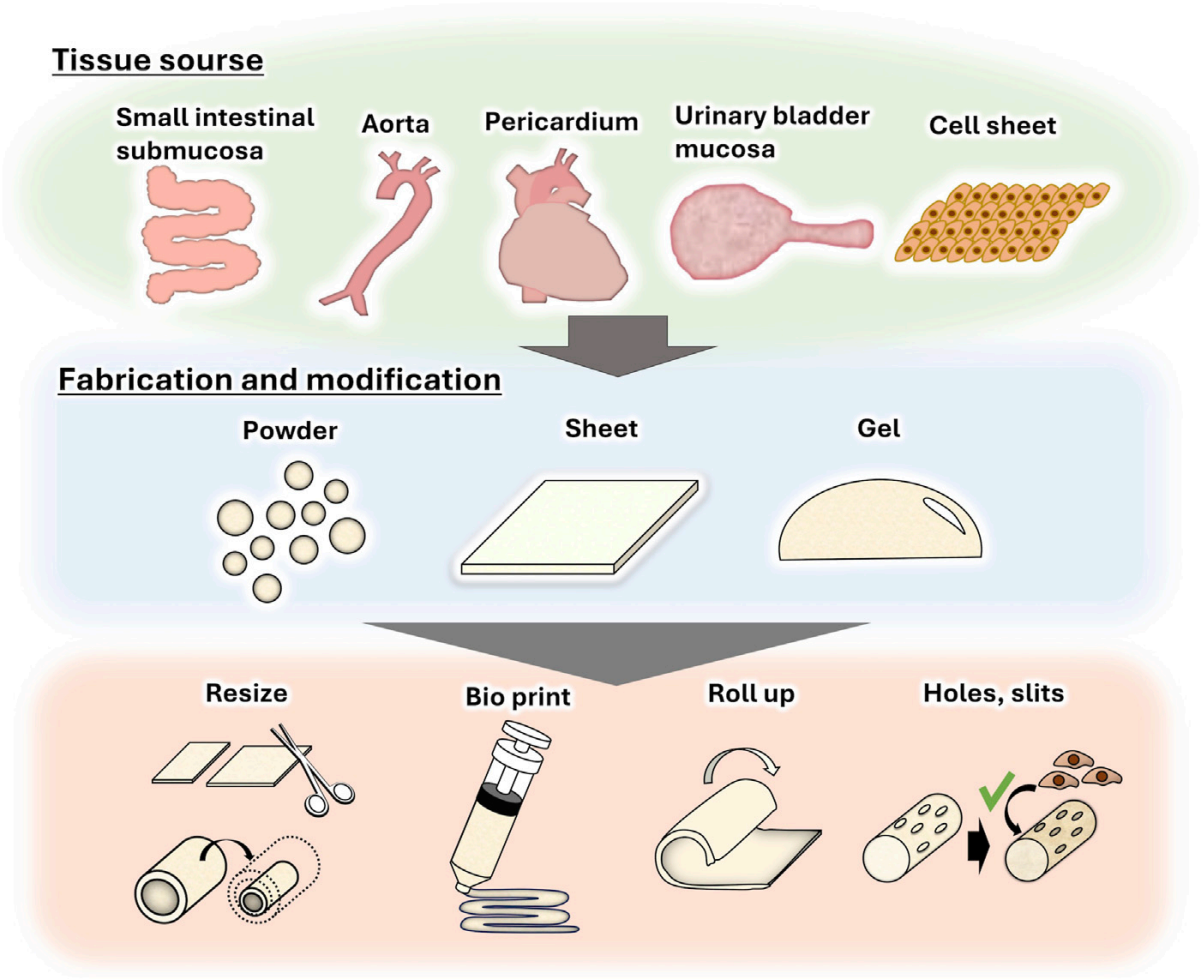
Fabrication and modification of decellularized tissues for further application.

Because of the high biological acceptability of decellularized tissues, they are often fabricated into powders (Edgar et al., 2018), sheets (Ning et al., 2017), and gels (Spang and Christman, 2018) for use in a wide range of applications (Figure 2). For example, decellularized tissue powder is used for soft tissue wound healing (Wolf et al., 2012) and acute myocardial infarction treatment (Tabuchi et al., 2015). We also reported that a sheet of decellularized aortic intermediates could be transformed into a tube and used as an alternative small-diameter vascular graft (Negishi et al., 2017b). Recently, decellularized powder and its solution were used as bio-ink for three-dimensional (3D) tissue printing (Abaci et al., 2020; Pati et al., 2014) to create tissues that mimic natural tissue. In terms of tendon and ligament regeneration, although decellularization has been effectively achieved for tight, rigid, and dense tissues, such as ligaments and tendons, recipient cells cannot easily infiltrate these tissues (Schulze-Tanzil et al., 2012; Tozer and Duprez, 2005). To resolve this issue, decellularized tendons are fabricated by creating holes and slits to promote cell infiltration while maintaining their shape (Tozer et al., 2005). In addition, a decellularized tendon sheet has been used for orthotopic tendons (Ning et al., 2017). Strategies such as the use of bovine pericardium, a membrane-like tissue, as a shoulder rotator cuff patch (Shim et al., 2022), and 3D rolling of the pericardium into a ligament-like tissue (Suzuki et al., 2022) are also being studied.

## 7 Soft-hard interregional regeneration

**FIGURE 3:**
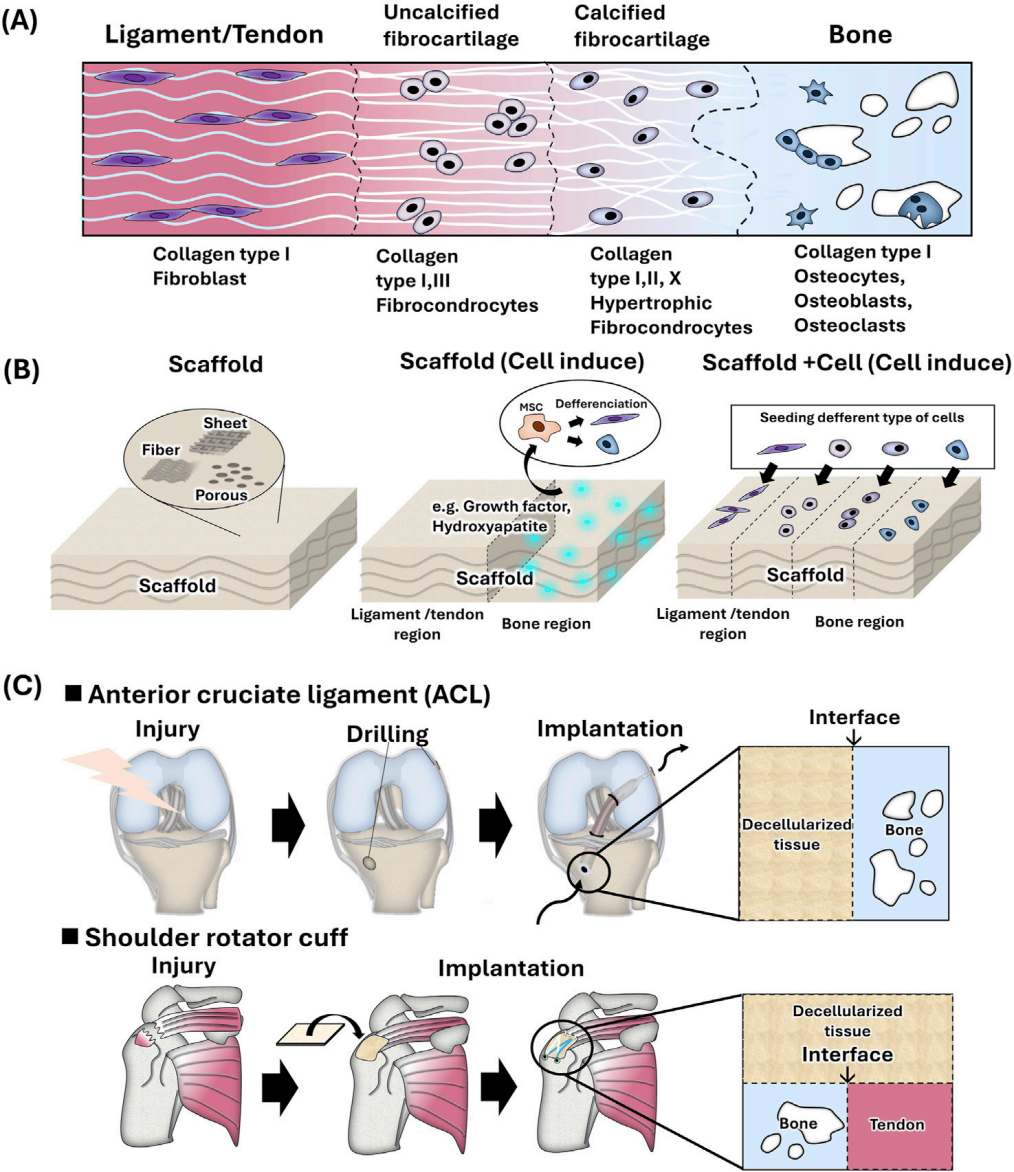
**(A)** Schematic of bone-to-soft interregional tissue in the human ligament/tendon. **(B)** Strategies for mimicking soft-hard interregional tissue using tissue engineering techniques. **(C)** Hard-soft tissue interface of tendon and ligament reconstruction using decellularized tissue.

In the human body, interregional tissues, such as tendon, ligament, and cartilage, are present between soft and hard tissues. These boundary tissues have complex structures with gradations in cell morphology, tissue composition, calcification, structure, and mechanical properties owing to the seamless connections between hard and soft tissues. Enthesis, which is a joint part of the tendon/ligament and bone is referred to as the interregional tissue. Enthesis is distinguished into four regions: tendon/ligament region, uncalcified region, calcified region, and bone region. The ligament/tendon region is composed mainly of type I collagen and fibroblasts. Non-calcified regions consist of type II and type III collagen and fibrochondrocytes, and calcified regions consist of type II and type X collagen and hypertrophic fibrochondrocytes. The bone region consists of type I collagen, carbonate apatite, hydroxyapatite, and osteocytes (Seidi et al., 2011; Figure 3A). Using tissue engineering technology, polymeric scaffold having two or multiple layers are used to mimic these four regions (Seidi et al., 2011; Lei et al., 2021; Pitta Kruize et al., 2023; Figure 3B). Fibrous or porous scaffolds are mainly used. Since tendons and ligaments are fiber tissues, the fabrication of fiber structures using electrospinning and the design of different fiber alignments (aligned or random) for each layer have been investigated widely in order to mimic fiber structure as polymeric scaffold (Samaveid et al., 2014; Liu et al., 2017). Calcification of polymer scaffolds has also been studied to mimic the composition of bone region. The bone region was prepared by alternative soaking method or the use of a nano-sized hydroxy apatite (Kim et al., 2014; Li et al., 2016; Li et al., 2017 (nHAP)). On the calcified scaffold, the affinity to bone-related cells, osteoblast, and osteocytes, has been showed *in vitro* and the bone was formed *in vivo*, while the affinity to fibroblast and fibrous formation was showed on the fibrous region. Also, the biological active molecules, BMP-2, PDGF, and TGF beta, were incorporated to the bone region of scaffold to induce the bone formation (Wei et al., 2020; Min et al., 2014). Also, cell-based approaches have been investigated, such as seeding different cell types before implantation (Lyu, et al., 2020). Thus, it is important to provide a microenvironment suitable for each bone and fibrous region.

In clinical surgery, the gold standard for anterior cruciate ligament (ACL) reconstruction is to transplant an autologous tendon such as a patellar tendon and hamstrings tendon after drilling a hole in the femoral or tibial head. However, autologous tendon grafting is highly invasive, and postoperative pain has been reported at the site of graft harvest (Kartus et al., 2001). Shoulder rotator cuff repair has not been successful despite advances in surgical techniques and changes in postoperative rehabilitation strategies. Rotator cuff repairs result in a high rate of postoperative tendon re-tears (Galatz et al., 2004). Most surgeries directly implant replacement tissue without soft-hard inter region and it is considered to induce the effective repair by using replacement tissue with soft-hard inter region. Therefore, from a tissue engineering perspective, it may be necessary to design inter regions with soft tissue affinity and hard tissue affinity in order to improve surgery outcomes (Figure 3C).

A few uses of decellularized tissue have been attempted to soft-hard interregional regeneration. It has reported tissue-based regeneration, such as decellularization in the bone-ligament-bone interface region for grafting (Uquillas et al., 2022; Harrison et al., 2005; Woods and Gratzer, 2005) and decellularized bovine pericardium patches (after cell seeding) for rotator cuff (bone and tendon) repair (Shim et al., 2022). To provide bone affinity with the tissue for ligament reconstruction, the mineralization of decellularized porcine pericardium (bone and ligament) (Suzuki et al., 2023) and decellularized bovine tendon (bone and tendon) (Grue and Veres, 2020) has also been studied. In these studies, decellularized tissue is powdered, solubilized, and made into a gel to incorporate various cell types and create boundary regions (Yun et al., 2023). Decellularized tendon-derived stem cell sheets have been used for cell-based regeneration. Wrapping tendon grafts with tendon-derived stem cell sheets promotes graft healing after ACL reconstruction. These are thought to promote osteogenesis and angiogenesis by vascular endothelial growth factor (VEGF) via the modulation of macrophage polarization and matrix metallo protease/tissue inhibitor of metalloproteinases (MMP/TIMP) expression, as well as the physical protection of the tendon graft (Yao et al., 2023). It has also been used to prepare tendon tissue *in vitro* using stem cells (iPS) and are decellularized to prepare tendons composed of iPS-derived tendons. Transplantation of iPS-derived tendons into a mouse Achilles tendon rupture model resulted in host-derived cell infiltration, and improved histological scores and biomechanical properties (980 Tsutsumi et al., 2022). From described above, many strategies related to decellularization technology has been proposed to regenerate soft-hard inter region, and in the future, it is expected that more research on region regeneration in various parts of body will be conducted.

## 8 Soft-hard interregional regeneration in dentistry

The periodontal ligament (PDL) is a fibrous tissue between the tooth and mandibular bone, into which Sharpey’s fibers are inserted and fixed into tooth and bone. It has a chewy texture and plays an important role in preventing periodontal disease. Recently, several studies on the reconstruction of the PDL have been conducted. A decellularized mandibular bone with a periodontal ligament matrix has been prepared through the extraction of mandibular bone with teeth, and the recellularization of the periodontal matrix (Nakamura et al., 2019; Son et al., 2019). It has also been reported to have a high affinity for decellularized mandibular bone with a periodontal ligament matrix for dental titanium implants (Yamada et al., 2022). Basic studies have also been conducted using the bovine pericardium as a periodontal ligament (affinity with PDL cells). Although tissue-based regeneration using decellularized tissue is the main method for tendon and ligament regeneration, as described above, cell-based regeneration has also been evaluated for PDL reconstruction due to fewer limitations related to mechanical strength. Since PDL is made from thin and breakable tissue, it is difficult to harvest PDL from living tissue intact. Therefore, not only tissue-regeneration but also a wide variety of cell based-regeneration was investigated. In a previous study, PDL cells were cultured and harvested as PDL sheets, facilitating the use of PDL cells without decellularization (Raju et al., 2020). The sheet of PDL cells was also decellularized and the remaining ECM was applied as a PDL scaffold (Heng et al., 2016; Farag et al., 2017; Farag et al., 2018a; Farag et al., 2018b). A previous study proposed that decellularized membrane tissue can be used to produce a PDL cell sheet (Iwasaki et al., 2019). Also, PDL regeneration strategies depend on the target of application, such as dental implants or periodontal defects. For dental implants, PDL cell sheets and gels are commonly used strategies. For periodontal defects, since not only PDL, but also tissue and bone regeneration are required, guided tissue regeneration (GTR) or guided bone regeneration (GBR) method using sheets have been investigated. Future developments are expected as decellularized tissues with various shapes and components are developed for periodontal disease and implant applications.

## 9 Discussion

For about two decades, in the orthopedic field, the decellularized tendon and ligament have been developed as an alternative ligament. Many chemical and physical decellularization methods are proposed, and optimized without decreasing the component, histological structure, and mechanical properties of tissue. The decellularized tendon and ligament showed good biocompatibility *in vitro* and *in vivo*. However, several issues for regeneration of ligament are remained: one is recellularization of decellularized tendon and ligament. We discuss it in the physical and biological aspects as below. As physical aspect, the tendon and ligament are mainly composed of collagen fibers with high-density and tightly, and the infiltration of host cell is difficult and takes time. The recellularization of host cell around the surface and the end of decellularized tendon and ligament was achieved relative-effectively, while the recellularizing in center of the decellularized tendon and ligament is still not enough. So, to enhance the cellular infiltration into the decellularized tendon and ligament, several fabrications such as making hole and slicing have been proposed. Further progress of fabrication method of decellularized tissue was expected in the viewpoint of wide application of decellularized tissues. As biological aspect, the immunological reaction to decellularized tendon and ligament is not still clear not only for decellularized tendon and ligament but also for other decellularized tissues. Generally, the acute immune responses to decellularized tissue was relatively low although the mechanism is still unknown. Recently, the macrophage polarization of M1 (inflammatory response) and M2 (anti-inflammatory response), which is a biological response to foreign materials, is considered as a key process, and the early switching of M1 to M2 of macrophage is considered to induces the tissue regeneration. It is needed to investigate the host cell behaviors relating the macrophage polarization, and to find the key factors of decellularized tissue controlling the macrophage polarization in the future. Second issue is regeneration of the soft-hard interregional tissue, such as bone-ligament, bone-tendon. The soft-hard interregional tissue has feature changing of component and structure gradually. The several decellularized gradient tissues, bone-ACL-bone, bone-cartilage, were proposed to alternative use, but the reports are a few and the regeneration mechanism is not understood. This may cause mismatch of the decellularized bone-ACL-bone to the established surgical operation protocol of ACL replacement, or the size and supply of decellularized bone-to bone. So, we have proposed a fabrication method of roll-formation and mineralization of the decellularized pericardial membrane to be used for the regeneration of soft-hard interregional tissues including bone-ACL and bone-PDL. In this method, the shape, size, and mineralization could be adjusted to the surgical operation. Also, the creation of soft-hard interregional tissue by 3D bioprinting have been attempted to be used for tissue regeneration and to understand the mechanism of regeneration process. In the dental field, the regeneration of periodontal ligament, the soft-hard interregional tissue, using GBR and GTR methods have been established, the introduction of decellularization techniques to the regeneration of periodontal ligament tissue is recent. Two strategies have been proposed mainly: tissue-based regeneration and cell-based regeneration. Both strategies are explored to be applied for the treatment of periodontal diseases. Future developments are expected as decellularized tissues with various shapes and components are developed for periodontal disease and implant applications.
